# MEA6 Deficiency Impairs Cerebellar Development and Motor Performance by Tethering Protein Trafficking

**DOI:** 10.3389/fncel.2019.00250

**Published:** 2019-06-11

**Authors:** Xin-Tai Wang, Xin-Yu Cai, Fang-Xiao Xu, Lin Zhou, Rui Zheng, Kuang-Yi Ma, Zhi-Heng Xu, Ying Shen

**Affiliations:** ^1^Department of Neurobiology, NHC and CAMS Key Laboratory of Medical Neurobiology, Zhejiang University School of Medicine, Hangzhou, China; ^2^State Key Laboratory of Molecular Developmental Biology, CAS Center for Excellence in Brain Science and Intelligence Technology, Institute of Genetics and Developmental Biology Chinese Academy of Sciences, Beijing, China

**Keywords:** MEA6, motor learning, Slit2, dendrite self-crossing, Fahr’s syndrome

## Abstract

Meningioma expressed antigen 6 (MEA6), also called cutaneous T cell lymphoma-associated antigen 5 (cTAGE5), was initially found in tumor tissues. MEA6 is located in endoplasmic reticulum (ER) exit sites and regulates the transport of collagen, very low density lipoprotein, and insulin. It is also reported that MEA6 might be related to Fahr’s syndrome, which comprises neurological, movement, and neuropsychiatric disorders. Here, we show that MEA6 is critical to cerebellar development and motor performance. Mice with conditional knockout of MEA6 (Nestin-Cre;MEA6^F/F^) display smaller sizes of body and brain compared to control animals, and survive maximal 28 days after birth. Immunohistochemical and behavioral studies demonstrate that these mutant mice have defects in cerebellar development and motor performance. In contrast, PC deletion of MEA6 (pCP2-Cre;MEA6^F/F^) causes milder phenotypes in cerebellar morphology and motor behaviors. While pCP2-Cre;MEA6^F/F^ mice have normal lobular formation and gait, they present the extensive self-crossing of PC dendrites and damaged motor learning. Interestingly, the expression of key molecules that participates in cerebellar development, including Slit2 and brain derived neurotrophic factor (BDNF), is significantly increased in ER, suggesting that MEA6 ablation impairs ER function and thus these proteins are arrested in ER. Our study provides insight into the roles of MEA6 in the brain and the pathogenesis of Fahr’s syndrome.

## Introduction

Meningioma expressed antigen 6 (MEA6), also known as cutaneous T cell lymphoma-associated antigen 5 (CTAGE5), was found in tumor tissues and usually considered as a risk gene for the cancer (Heckel et al., 1997; Comtesse et al., 2002; Kalniņa et al., 2008). Recent studies show that MEA6 is also expressed in secretary organs (such as liver and pancreas), brain, spleen, lung, and intestine (Wang et al., 2016; Fan et al., 2017). Structurally, MEA6 is a coil-coiled protein with its main structure containing one transmembrane domain, two coiled-coil domains, and one proline-rich domain (Wang et al., 2016). Functionally, MEA6 localizes in endoplasmic reticulum (ER) exit site and interacts with Golgi organization 1 (TANGO1) and Sec proteins to regulate the secretion of collagen, a large cargo (Saito et al., 2011, 2014). Surprisingly, recent studies demonstrate that MEA6 also regulates the transport of small cargos. MEA6 is involved in the transport and secretion of very low-density lipoprotein (VLDL) through the assembly of coat protein complex II (COPII) complex in the liver and insulin secretion through interacting with Sec22 in the pancreas (Wang et al., 2016; Fan et al., 2017).

Meningioma expressed antigen 6 is highly expressed in the central nervous system (CNS), however, its functions in cellular component trafficking of neurons, brain development and animal behaviors are poorly understood. Utilizing conditional knockout mice of MEA6 driven by Nestin-Cre, Zhang et al. (2018) recently show that MEA6 is important for brain development and function. The deletion of MEA6 in the brain leads to reduced brain size, early death of mice, and behavioral defects, including abnormal limb-clasping reflex and impaired motor performance in the rotarod test (Zhang et al., 2018). These phenotypes may be ascribed to the deficits in neural development, since the dendrite outgrowth and branching, spine formation and maintenance, and astrocytic activation are damaged in the cerebral cortex of Nestin-Cre;MEA6^F/F^ mice (Zhang et al., 2018). Nevertheless, it is not yet answered how MEA6 affects the development of the cerebellum. Particularly, a loss-of-function mutation of MEA6 (P521A) might be associated with Fahr’s syndrome (Lemos et al., 2011), an inheritance neurological disorder comprising neurological, movement, and neuropsychiatric disorders (Oliveira et al., 2007). The patients with Fahr’s disease show severe degeneration in brain regions controlling movement and exhibit unsteady gaits (Moskowitz et al., 1971; Geschwind et al., 1999; Saleem et al., 2013). Thus, it is attractive to investigate how MEA6 knockout causes abnormal cerebellar development and dysfunctional motor behaviors, which will lead to more insights of the pathogenesis of Fahr’s syndrome.

Here, we set out to establish the contribution of MEA6 in cerebellar development and motor functions using conditional approaches to generate specific deletion. We demonstrate that Nestin-Cre;MEA6^F/F^ mice have defects in cerebellar development and motor performance. In contrast, mice with Purkinje cell (PC)-specific deletion of MEA6 (pCP2-Cre;MEA6^F/F^) have normal lobular formation and walking gait, but display impaired self-avoidance of PC dendrites and motor learning. We further found that the expression of several molecules critical to cerebellar development, including Slit2, brain derived neurotrophic factor (BDNF) and Semaphorin 3A, is significantly increased in ER, suggesting that these proteins are tethered in ER, but not functionally expressed, by MEA6 ablation.

## Materials and Methods

### Animals

All experiments were approved by the Animal Experimentation Ethics Committee of Zhejiang University and specifically designed to minimize the number of animals used. The construction of MEA6^F/F^ mice was described previously (Wang et al., 2016). Nestin-Cre;MEA6^F/F^ (N-Cre;MEA6^F/F^) and pCP2-Cre;MEA6^F/F^ (P-Cre;MEA6^F/F^) mice were obtained by crossing MEA6^F/F^ mice with Nestin-Cre and pCP2-Cre mice (Zhou et al., 2015, 2017), respectively. The resulting offspring were genotyped using PCR of genomic DNA, which were: MEA6 floxP fragment, F: 5′-GAC ACT TGA CCC CTC CTC TCC-3′; R: 5′-AAC GGC TCA TGC TTG CTA ACC-3′; Nestin-cre, F: 5′-GCG GTC TGG CAG TAA AAA CTA TCT-3′; R: 5′-GTG AAA CAG CAT TGC TGT CAC TT-3′; pCP2-cre, F: 5′-TGC CAC GAC CAA GTG ACA GCA ATG-3′; R: 5′-ACC AGA GAC GGA AAT CCA TCG CTC-3′. Mice were kept at the Experimental Animal Center of Zhejiang University under temperature-controlled condition on a 12:12 h light/dark cycle. All experiments were performed blind to genotypes in age-matched littermates of either sex.

### Antibodies and Reagents

The antibody against γ-protocadherin (γ-Pcdh) was from Synaptic Systems (Gottingen, Germany). The antibody against VDAC was from Cell Signaling (Danvers, MA, United States). Antibodies against GAPDH, PSD95, Rab11, and TrkB were from Millipore (Billerica, MA, United States). Antibodies against Slit2 and γ-adaptin were was from Proteintech (Rosemont, IL, United States). Antibodies against MEA6 and calbindin were from Sigma (St. Louis, MO, United States). Antibodies against Bip, PDI, Robo2, and Semaphorin 3A were from Abcam (Cambridge, United Kingdom). Antibodies against β-actin, β-tubulin, YY1, and BDNF were from Santa Cruz (Dallas, TX, United States). Goat anti-mouse and anti-rabbit IgG horseradish peroxidase-conjugated were from Thermo Fisher Scientific (Waltham, MA, United States). The alexa fluor-conjugated secondary antibody was from Invitrogen (Carlsbad, CA, United States). Protease inhibitor cocktail was from Roche (Mannheim, Germany). Other chemicals were from Sigma unless stated otherwise.

### Purification of ER

Endoplasmic reticulum fractions were purified according to Hammond et al. (2012). In brief, cerebellar tissues were homogenized in isotonic extraction buffer (10 mM HEPES, 250 mM sucrose, 25 mM KCl, 1 mM EGTA; pH 7.8) supplemented with protease inhibitors. A centrifugation (700 × *g*; 10 min) was used to remove nuclei and large cellular debris (P1). A subsequent 15,000 × *g* (10 min) of supernatant (S1) was performed to pellet mitochondria (P2). The resulting supernatant (S2) was loaded onto a three-layered sucrose gradient (2.0, 1.5, and 1.3 M) and centrifuged at 126,000 × *g* for 70 min on a ultracentrifuge. The upper supernatant was collected (S3) and the white band between the top and 1.3 M-sucrose layers was collected (P3), which was gently mixed by inversion with ice cold MTE solution (270 mM D-mannitol, 10 mM Tris-base, 0.1 mM EDTA; pH 7.4 with HCl) supplemented with protease inhibitors. This mixture was centrifuged at 126,000 × *g* for 45 min resulting in a large and translucent pellet (P4).

### RT-PCR

The total RNA of the cerebellum was isolated using TRIzol Reagent (Thermo). cDNA was synthesized by reverse transcription using oligo (dT) as the primer using Revert Aid First Strand cDNA synthesis Kit (Thermo). For single-cell analysis, the contents of individual PCs (P21) were harvested as described in previous work (Zhou et al., 2015, 2017). The tip of a conventional patch-clamp pipette was placed tightly on the soma of a selected PC and a gentle suction was applied to the pipette. After complete incorporation of the soma, the negative pressure was released and the pipette was quickly removed from the bath. The harvested contents were subjected to RT-PCR using OneStep Kit (Qiagen, Germany). Forward (F) and reverse (R) primers used for amplification were as follows: MEA6, F: 5′-GTT GAA GGA TCA CAA ATA TC-3′; R: 5′-TCC TTT TTG AAA TAT CAG CC-3′; calbindin, F: 5′-GGC TTC ATT TCG ACG CTG AC-3′; R: 5′-ACG TGA GCC AAC TCT ACA ATT C-3′; GAPDH, F: 5′-GGT GAA GGT CGG TGT GAA CG-3′; R: 5′-CTC GCT CCT GGA AGA TGG TG-3′.

### Western Blot

The protein concentration was determined using BCA protein assay. Equal quantities of proteins were loaded and fractionated on SDS–PAGE, transferred to PVDF membrane (Immobilon-P, Millipore), immunoblotted with antibodies, and visualized by enhanced chemiluminescence (Thermo Fisher Scientific). The dilutions of antibodies were MEA6 (1:4,000), Slit2 (1:1,000), Robo2 (1:1,000), β-tubulin (1:2,000), GAPDH (1:20,000), γ-Pcdh (1:2,000), BDNF (1:1,000), TrkB (1:1,000), PDI (1:1,000), Bip (1:5,000), γ-adaptin (1:1,000), YY1 (1:300), PSD95 (1:40,000), VDAC (1:1,000), Rab11 (1:1,000), Semaphorin 3A (1:1,000), and secondary antibodies (1:10,000). Film signals were digitally scanned and quantitated using ImageJ 1.42q (NIH).

### Immunohistochemistry

Sagittal frozen sections (30 μm) were cut and placed in blocking solution for 1 h at room temperature (RT). After washing with PBS, sections were incubated with primary antibodies overnight at 4°C and incubated with secondary antibodies for 1 h at RT. Primary antibody dilutions used for immunohistochemistry were calbindin (1:500), NeuN (1:500), and MEA6 (1:250). Alexa Fluor 488-conjugated goat anti-mouse IgG, Alexa Fluor 594-conjugated goat anti mouse IgG and Alexa Fluor 488-conjugated goat anti-rabbit IgG antibody were diluted at 1:1,000. All antibodies were diluted in PBS containing 1% BSA and 1% normal goat serum. To offset weak staining ability of MEA6 antibody, we enhanced the excitation of confocal microscope (emission wavelength: 525 nm; excitation wavelength: 488 nm; pinhole radius: 22.99 μm; lser power: 13.84%, high voltage: 123).

### H&E Staining

H&E staining was performed by using H&E staining Kit (Beyotime, Shanghai, China) (Xie et al., 2015). Sagittal cerebellar slices (30 μm) were immersed in hematoxylin staining solution for 5–10 min, rinsed with distilled water, and immersed in eosin staining solution for 2 min. The sections were then rinsed with distilled water, dehydrated in ethanol, and cleared in xylene. Images of cerebellar cortex were captured using a light microscope. For quantification, total cerebellar area, the thickness of lobule, the thicknesses of granule cell layer and molecular layer were calculated using ImageJ. The maximum width of a cerebellar lobule was defined as its thickness. The intermediate point of the long axis of a lobe was selected to measure the thicknesses of granule cell and molecular layers. The experimenter was blind to mouse genotypes during the measurement.

### Nissl Staining

Nissl staining was performed using Nissl staining Kit (Beyotime) (Xie et al., 2015). Sagittal cerebellar slices (30 μm) were immersed in Nissl staining solution for 5 min, rinsed with distilled water, dehydrated in ethanol, and cleared in xylene. Images of the cerebellar cortex were captured using a light microscope. The quantification of cerebellar area, lobular thickness, and the thicknesses of granule cell and molecular layers in Nissl staining were measured as same as those in H&E staining.

### Analysis of PC Dendrites

sUnder the anesthesia with an intraperitoneal injection of pentobarbital (30 mg/kg), mice (P21) were placed in a stereotaxic apparatus (Stoelting, Wood Dale, IL, United States) and multiple holes (∼1 × 1 mm^2^) were opened above cerebellar vermis. According to stereotaxic coordinates (bregma: anteroposterior, −5.5 mm; mediolateral, −0.5 mm; dorsoventral, −1.2 mm), a Semliki Forest virus (SFV) solution (500 nl; 2.3E6 FFU/ml) containing mCherry or GFP was injected into lobules IV-V with the pressure of a glass pipette (30 μm in diameter). After injection, animals were put on an electric blanket for more than 30 min for recovery from the anesthesia, and then returned to their cages.

Injected animals were decapitated within 18 h after viral infection and cerebella were sectioned (20 μm). Optical sections were collected on a confocal microscope and analyzed using Imaris 9.0 (Bitplane, Concord, MA, United States). The z-distance between crossing branches was calculated from 3D reconstructions. Any two branches crossing over each other were marked and counted manually in z-projections. The number of self-crossing, total number of dendritic branches, and total dendrite length were calculated from 3D reconstruction according to previous work (Gibson et al., 2014). Total dendrite area was calculated from 2D reconstruction using Image-Pro Plus 6.0 (Media Cybernetics, Rockville, MD, United States).

### Rotarod Test

Rotarod test was performed as previously described (Zhou et al., 2017). After the habituation to rotarod, N-Cre;MEA6^F/F^ mice (P19-21) and P-Cre;Mea6^F/F^ mice (2 month) were tested twice a day at a time interval of 8 h for 4 consecutive days. In each session, the velocity of rotation increased at a constant acceleration of 9 rpm/min starting from 5 rpm. Given the short lifetime, N-Cre;Mea6^F/F^ mice at P19-21 were put on the rotarod at a fixed 20 rpm and the spent time on the rotarod was measured.

### Elevated Beam Test

This test was performed according to previous work (Hartmann et al., 2014; Zhou et al., 2017). The movement of mice on a round plastic beam (length 50 cm and diameter 1 cm) 40 cm above a surface with bedding was recorded and analyzed. The percentage of steps with hindpaw slips during runs on the beam was calculated.

### Foot Print Test

To evaluate mice’s walking gait we used footprint test according to previous work (Wang et al., 2017). Mice hindpaws were painted with non-toxic ink, and they were allowed to freely traverse a clear plexiglass tunnel (100 × 10 × 10 cm), with a sheet of white absorbent paper (100 × 10 cm) placed at the bottom of the track and a darkened cage at the end of the tunnel to encourage the mouse to run toward a dark and safe environment. The resulting tracks provided the spatial relationship of consecutive footfalls, from which the stride length and stance width were measured. Measurements for three-step cycles were averaged, considering a cycle as the distance from one pair of hind prints to the next. Footprints at the start and the end of the tunnel were excluded from the analysis as they corresponded to the initiation and termination of the movement.

### Statistical Analysis

Data were analyzed using GraphPad Prism 6.0 (GraphPad Software, San Diego, CA, United States), Excel 2003 (Microsoft, Seattle, WA, United States), and Igor Pro 6.0 (Wavemetrics, Lake Oswego, OR, United States). Data analysts were blind to experimental conditions until the data were integrated. Standard deviations for controls were calculated from the average of all control data. Statistical difference was determined using two-sided unpaired Student’s *t*-test. The accepted level of significance was *p* < 0.05. *n* represents the number of preparations, cells, or animals. Data are presented as mean ± SEM.

## Results

### MEA6 Deletion in the Cerebellum Impairs Motor Performance

**FIGURE 1:**
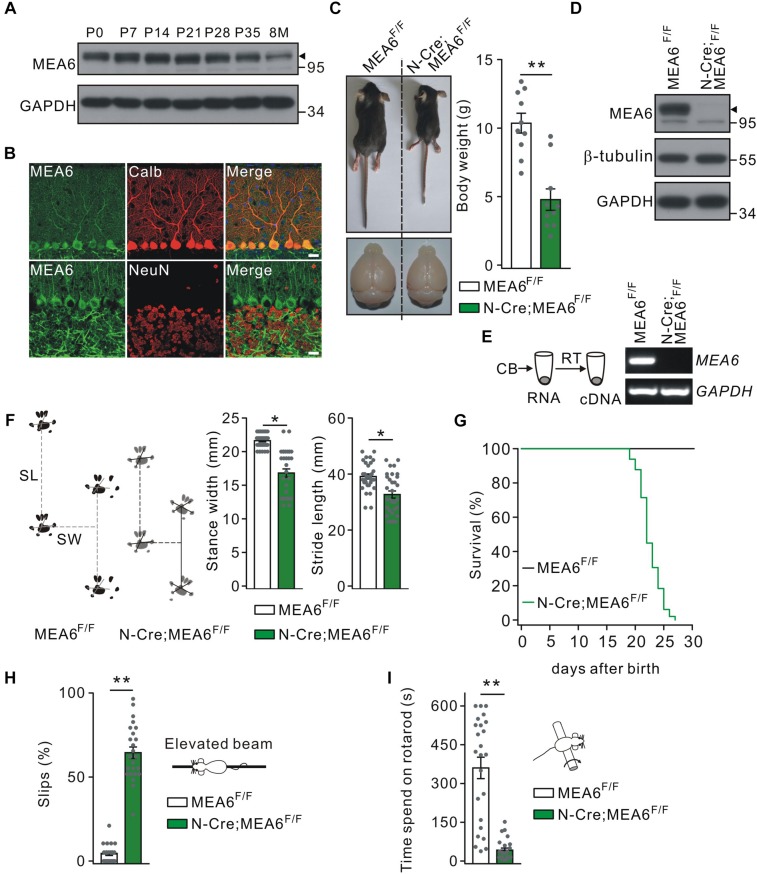
Nestin-Cre;MEA6^F/F^ mice display defects in motor performance. **(A)** MEA6 expression in the mouse cerebellum at different stages. Equal amounts of protein from cerebellar tissues were loaded onto SDS–PAGE and analyzed by Western blots using antibodies against MEA6 and GAPDH. The black triangle shows the bands of MEA6 protein. The experiment was performed three times. **(B)** Immunohistochemical staining for MEA6 (green), calbindin (Calb, red), and NeuN (red) in the cerebella from control mice. Scale bars: 20 μm. **(C)** The pictures of bodies and brains of MEA6^F/F^ and N-Cre;MEA6^F/F^ at P21. Average body weights were 10.4 ± 0.8 g (MEA6^F/F^; *n* = 10 mice) and 4.8 ± 1.1 g (N-Cre;MEA6^F/F^; *n* = 10 mice). Gray dots indicate individual data points. Unpaired *t*-test, ^∗∗^*p* < 0.01. **(D)** Western blot detection of MEA6 expression in MEA6^F/F^ and N-Cre;MEA6^F/F^ mice (P21). The black triangle shows the band of MEA6 protein. **(E)** The cerebella (CB) of MEA6^F/F^ and N-Cre;MEA6^F/F^ mice (P21) were extracted in centrifuge tubes and subjected to RT-PCR. The electrophoresis of MEA6 (157 bp) and GAPDH (233 bp) is show in the right (*n* = 5 trials from one pair of mice). **(F)** Footprints of hind paws of MEA6^F/F^ and N-Cre;MEA6^F/F^ mice. The statistics shows abnormal gait with a shorter stride width (SW) and stance length (SL) in N-Cre;MEA6^F/F^ (SW: 16.8 ± 0.8 mm, unpaired *t*-test, ^*^p < 0.05. SL: 32.7 ± 1.3 mm, unpaired *t*-test, ^*^p < 0.05. *n* = 32 trials from 5 mice), compared to MEA6^F/F^ mice (SW: 21.6 ± 0.2 mm. SL: 39.1 ± 0.9 mm. *n* = 68 trials from 6 mice). Gray dots indicate individual data points. **(G)** Kaplan-Meier survival curves of MEA6^F/F^ (*n* = 45 mice) and N-Cre;MEA6^F/F^ mice (*n* = 49 mice). **(H)** The percentages of hindpaw slips during runs on an elevated horizontal beam. MEA6^F/F^: 4.3 ± 1.0% (*n* = 24 trials from 6 mice). N-Cre;MEA6^F/F^: 64.9 ± 4.8% (*n* = 24 trials from 6 mice). Gray dots indicate individual data points. Unpaired *t*-test, ^∗∗^*p* < 0.001. **(I)** The time spent on the rotarod with a fixed speed for MEA6^F/F^ and N-Cre;MEA6^F/F^ mice at P19-21. MEA6^F/F^: 359 ± 62 s (*n* = 24 trials from 6 mice); N-Cre;MEA6^F/F^: 41 ± 8 s (*n* = 24 trials from 6 mice). Gray dots indicate individual data points. Unpaired *t*-test, ^∗∗^*p* < 0.001.

**FIGURE 2:**
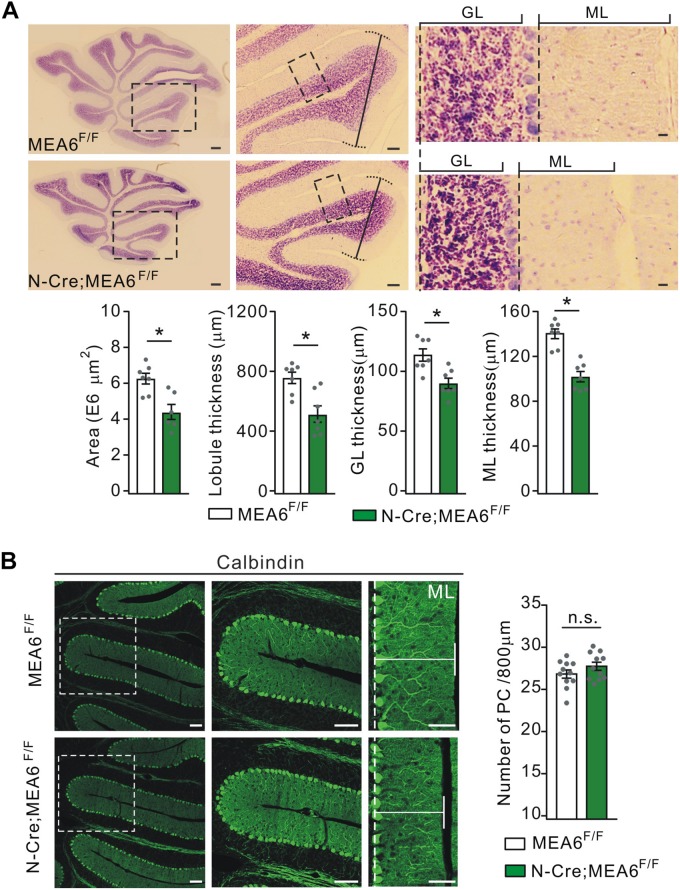
Shrunken lobules in N-Cre;MEA6^F/F^ mice. **(A)** The H&E staining of sagittal cerebellar sections from MEA6^F/F^ and N-Cre;MEA6^F/F^ mice at P25. The middle panel (Scale bars: 100 μm) is the higher magnification of left panel (Scale bars: 200 μm) and the right panel (Scale bars: 10 μm) is the higher magnification of middle panel, as indicated by black dashed boxes. Cerebellar area: 6.3 ± 0.4 E6 μm^2^ (MEA6^F/F^; *n* = 7 mice) and 4.5 ± 0.7 E6 μm^2^ (N-Cre;MEA6^F/F^; *n* = 6 mice), unpaired *t*-test, ^*^p < 0.05. Lobule III thickness: 758 ± 58 μm (MEA6^F/F^; *n* = 7 mice) and 518 ± 71 μm (N-Cre;MEA6^F/F^; *n* = 7 mice), unpaired *t*-test, ^*^p < 0.05. Thickness of granule cell layer (GL; lobule III): 110 ± 5 μm (MEA6^F/F^; *n* = 7 mice) and 90 ± 4 μm (N-Cre;MEA6^F/F^; *n* = 7 mice), unpaired *t*-test, ^*^p < 0.05. Thickness of molecular layer (ML; lobule III): 141 ± 4 μm (MEA6^F/F^; *n* = 7 mice) and 101 ± 4 μm (N-Cre;MEA6^F/F^; *n* = 7 mice), unpaired *t*-test, ^*^p < 0.05. Gray dots indicate individual data points. **(B)** The calbindin staining in MEA6^F/F^ and N-Cre;MEA6^F/F^ mice (P25), indicating that the number and arrangement of PCs are not changed by the deletion of MEA6. Average numbers of PCs per 800 μm were 26.8 ± 0.5 (MEA6^F/F^; *n* = 11 mice) or 27.7 ± 0.5 (N-Cre;MEA6^F/F^; *n* = 11 mice). Gray dots indicate individual data points. Unpaired *t*-test, *p* > 0.05 (n.s.).

The expression of MEA6 in the cerebellum was prominent and constant at postnatal stages but degraded a little after 8 month (Figure 1A). The expression pattern of MEA6 in the cerebellum was further examined using double immunostaining with cell-specific markers, showing that MEA6 was abundantly expressed in PCs and granule cells (Figure 1B). It should be noted that MEA6 antibody might be not so appropriate for staining because of its impurity. To assess the roles of MEA6 in the cerebellum, we generated conditional knockout of MEA6 in the brain by mating MEA6^F/F^ mice with N-Cre transgenic mice, which affected MEA6 expression in most neural cells, including PCs, granule cells and glia. Compared with the control MEA6^F/F^ mice, body weight and cerebellar size were significantly decreased in N-Cre;MEA6^F/F^ mice (Figure 1C). The knockout of MEA6 in N-Cre;MEA6^F/F^ mice was confirmed by Western blot (Figure 1D) and RT-PCR (Figure 1E) assays. N-Cre;MEA6^F/F^ mice showed abnormal limb-clasping reflex in tail suspension test, in consistent with previous work (Zhang et al., 2018), and walked abnormally with shorter hindlimb stride length and stance width (Figure 1F), which might be related to their smaller body size. In fact, all N-Cre;MEA6^F/F^ mice died before P28 (Figure 1G). Besides these degenerative phenotypes, we found that N-Cre;MEA6^F/F^ mice displayed defective motor performance: (1) They performed poorly when walking on a narrow elevated beam with a remarkably higher number of hind-paw slips (Figure 1H); (2) They spent dramatically less time on the rotating rod in rotarod test (Figure 1I). These results indicate that MEA6 plays important roles in brain development.

### MEA6 Deletion Reduces the Thicknesses of Lobules, Granule Cell Layer, and Molecular Layer

To examine the roles of MEA6 in the development of the cerebellum, the morphogenesis of N-Cre;MEA6^F/F^ cerebella (P25) was observed in sagittal sections. While H&E staining showed that folia formation was grossly normal, there was an apparent reduction in the area of whole cerebellum and the thickness of lobules (Figure 2A). The averages of both area and thickness of lobule III shrunk by almost 60% in N-Cre;MEA6^F/F^ mice compared to MEA6^F/F^ mice (Figure 2A). Another phenotype was that the thicknesses of granule cell layer (GCL) and molecular layer (ML) were much reduced (lobule III; Figure 2A), resulting in an increased cell density in GCL of N-Cre;MEA6^F/F^ mice.

Due to the remarkable expression of MEA6 in PCs (Figure 1B), we examined whether PC layer (PCL) is changed by the deletion of Mea6 in N-Cre;MEA6^F/F^ mice. Unexpectedly, calbindin staining showed no abnormality in PCL, as the number and arrangement of PCs were comparable between N-Cre;MEA6^F/F^ and MEA6^F/F^ mice (Figure 2B). Still, we found that the dendritic branch of PCs was much reduced (Figure 2B), consistent with the reduced thickness of ML shown in Figure 2A.

### PC-Specific Deletion of MEA6 Deletion Impairs Motor Learning but Not Walking Gait

**FIGURE 3:**
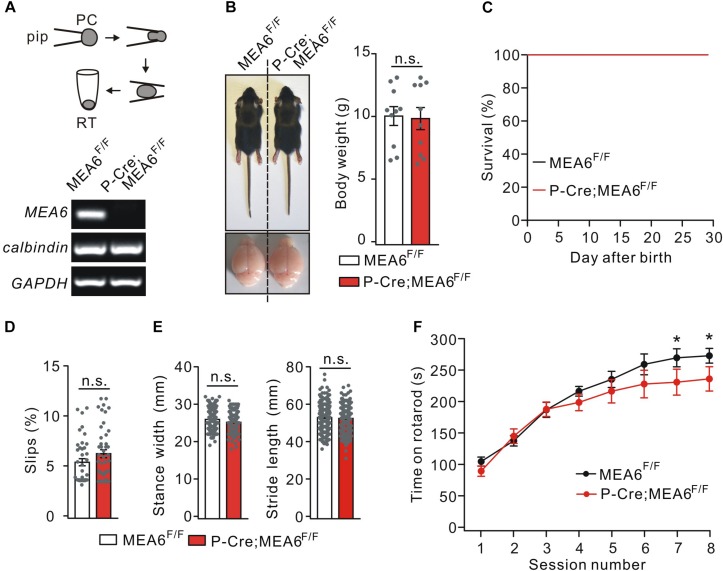
The deletion of MEA6 in PCs does not affect gait but impairs motor learning. **(A)** PC contents of MEA6^F/F^ and N-Cre;MEA6^F/F^ mice (P21) were harvested using glass micropipettes (pip, OD 2 μm) and placed in centrifuge tubes. The contents collected from 10 cells were then subjected to RT-PCR. The electrophoresis of MEA6 (157 bp), calbindin (184 bp), and GAPDH (233 bp) is show in the lower panel (*n* = 5 trials). **(B)** Pictures of bodies and brains of MEA6^F/F^ and P-Cre;MEA6^F/F^ at P21. Average body weights were 10.4 ± 0.8 g (MEA6^F/F^; *n* = 10 mice) and 10.2 ± 1.0 g (P-Cre;MEA6^F/F^; *n* = 10 mice). Gray dots indicate individual data points. Unpaired *t*-test. **(C)** Kaplan-Meier survival curves of MEA6^F/F^ (*n* = 15 mice) and P-Cre;MEA6^F/F^ mice (*n* = 15 mice). **(D)** The percentages of hindpaw slips during runs on an elevated horizontal beam. MEA6^F/F^: 5.3 ± 0.3% (*n* = 40 trials from 6 mice). P-Cre;MEA6^F/F^: 6.2 ± 0.5% (*n* = 45 trials from 6 mice). Gray dots indicate individual data points. Unpaired *t*-test, *p* > 0.05. **(E)** The statistics of footprints of MEA6^F/F^ and P-Cre;MEA6^F/F^ mice shows normal gait with unchanged stride width (SW) and stance length (SL) in P-Cre;MEA6^F/F^ (SW: 25.9 ± 0.4 mm, unpaired *t*-test, *p* = 0.69; SL: 45.4 ± 1.0 mm, unpaired *t*-test, *p* = 0.41; *n* = 172 trials from 16 mice), compared to MEA6^F/F^ mice (SW: 25.2 ± 0.2 mm; SL: 44.7 ± 0.8 mm; *n* = 197 trials from 16 mice). Gray dots indicate individual data points. **(F)** Time spent on the accelerating rotarod for MEA6^F/F^ (*n* = 8 mice) and P-Cre;MEA6^F/F^ mice (*n* = 8 mice) at P60. Unpaired *t*-test, ^*^p < 0.05.

To determine if motor defects in N-Cre;MEA6^F/F^ mice are caused by PC abnormality and to study the function of MEA6 in PCs, MEA6^F/F^ mice were mated with P-Cre transgenic mice. The knockout of MEA6 was confirmed by single-cell RT-PCR assay, showing that MEA6 mRNA was absent in P-Cre;MEA6^F/F^ mouse PCs (Figure 3A). Different from N-Cre;MEA6^F/F^ mice, P-Cre;MEA6^F/F^ mice had no defects in the body and brain size (Figure 3B). Meanwhile, the lifetime of P-Cre;Mea6^F/F^ mice was as long as that of MEA6^F/F^ mice (Figure 3C). Regarding motor behavior, P-Cre;MEA6^F/F^ mice did not show increased hind-paw slips during elevated beam test (Figure 3D) and had normal hindlimb stride length and stance width during the footprint test (Figure 3E), suggesting that PC deletion of MEA6 does not affect gait and that abnormal stance of N-Cre;Mea6^F/F^ mice is related to smaller body size. In the accelerating rotarod test, P-Cre;MEA6^F/F^ mice showed improvements as the sessions went by, but their time staying on the rod after six sessions was slightly reduced compared with that of controls (Figure 3F), implicating that MEA6 deficiency in PCs might impair motor learning.

### MEA6 Deficiency in PCs Does Not Affect Cyto-Architecture

**FIGURE 4:**
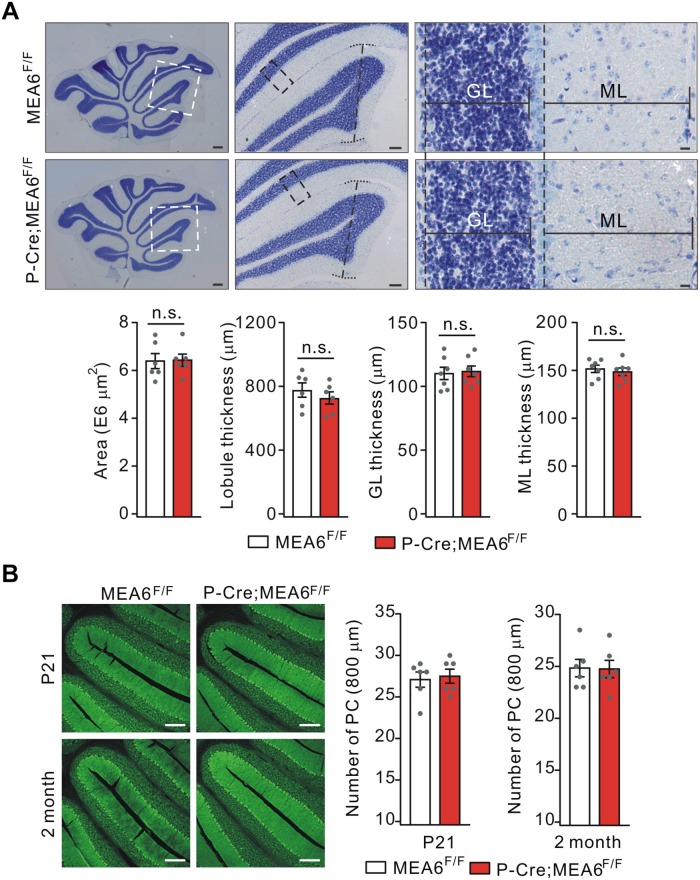
Lobular formation is normal in P-Cre;MEA6^F/F^ mice. **(A)** Nissl staining of cerebellar sections from MEA6^F/F^ and P-Cre;MEA6^F/F^ mice at P25. The middle panel (Scale bars: 100 μm) is the higher magnification of left panel (Scale bars: 200 μm) and the right panel (Scale bars: 10 μm) is the higher magnification of middle panel, as indicated by dashed boxes and lines. Cerebellar area: 6.4 ± 0.4 E6 μm^2^ (MEA6^F/F^; *n* = 6 mice) and 6.3 ± 0.3 E6 μm^2^ (P-Cre;MEA6^F/F^; *n* = 6 mice), unpaired *t*-test, *p* > 0.05. Lobule IV thickness: 774 ± 52 μm (MEA6^F/F^; *n* = 6 mice) and 729 ± 48 μm (P-Cre;MEA6^F/F^; *n* = 6 mice), unpaired *t*-test, *p* > 0.05. Thickness of granule cell layer (GL; lobule IV): 111 ± 5 μm (MEA6^F/F^; *n* = 7 mice) and 112 ± 5 μm (P-Cre;MEA6^F/F^; *n* = 7 mice), unpaired *t*-test, *p* > 0.05. Thickness of molecular layer (ML; lobule IV): 152 ± 5 μm (MEA6^F/F^; *n* = 7 mice) and 149 ± 5 μm (P-Cre;MEA6^F/F^; *n* = 7 mice), unpaired *t*-test, *p* > 0.05. Gray dots indicate individual data points. **(B)** The calbindin staining in MEA6^F/F^ and P-Cre;MEA6^F/F^ mice (P21 and P60), indicating that the number PCs is not changed by the deletion of MEA6. The average numbers of PCs per 800 μm were 27.1 ± 1.0 (MEA6^F/F^; *n* = 6 mice), 27.4 ± 0.9 (P-Cre;MEA6^F/F^; *n* = 6 mice), unpaired *t*-test, *p* > 0.05 at P21, and 24.8 ± 0.9 (MEA6^F/F^; *n* = 6 mice), 24.7 ± 0.9 (P-Cre;MEA6^F/F^; *n* = 6 mice), unpaired *t*-test, *p* > 0.05. Gray dots indicate individual data points. Scale bars: 200 μm.

We continued to examine the morphogenesis of P-Cre;MEA6^F/F^ cerebella at P21. Using Nissl staining of sagittal sections, we found that the cyto-architecture of cerebella in P-Cre;MEA6^F/F^ mice, including folia formation, the area of cerebella, the thickness of lobules, GCL thickness, and ML thickness, were not changed compared with those in MEA6^F/F^ mice, as measured in lobule IV (Figure 4A). We also examined PCL in P-Cre;MEA6^F/F^ mice at both P21 and P60 using calbindin staining. Similar to N-Cre;MEA6^F/F^ mice, we failed to find any abnormality in PCL at two ages, shown by the unchanged number and arrangement of PCs (Figure 4B). These data indicate that cerebellar cyto-architecture grossly keeps intact in P-Cre;MEA6^F/F^ mice.

### PC Deletion of MEA6 Leads to Self-Crossing of PC Dendrites

**FIGURE 5:**
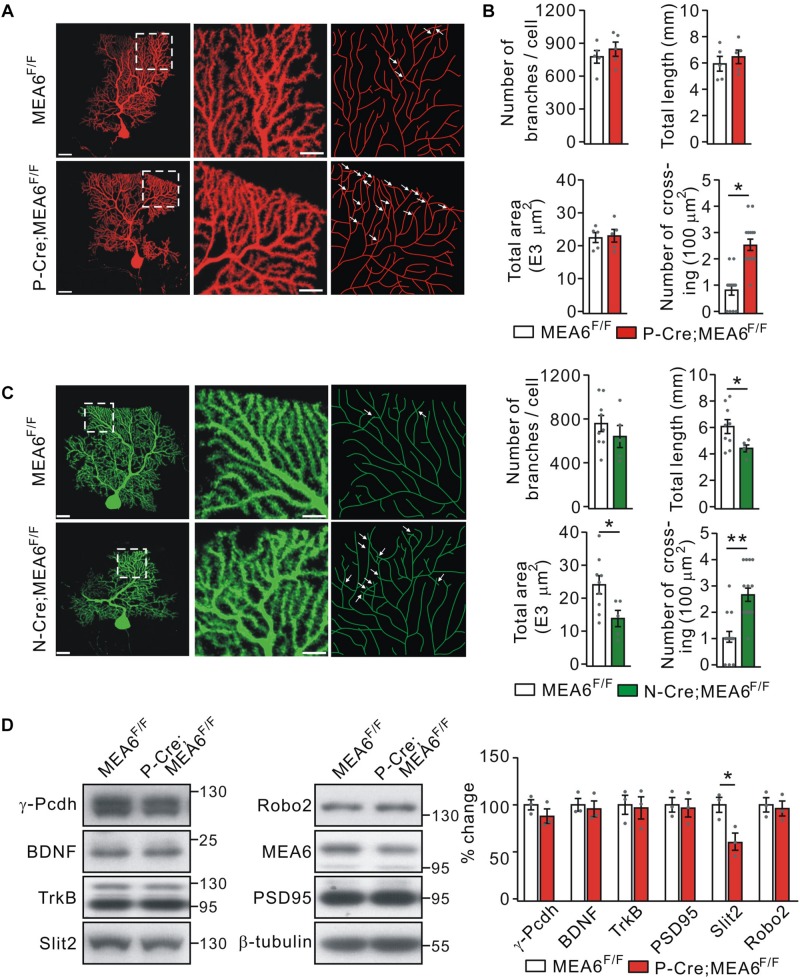
Purkinje cell deletion of MEA6 impairs dendrite self-avoidance. **(A)** PCs from MEA6^F/F^ and P-Cre;MEA6^F/F^ mice (P21) are shown by z-projections of confocal images (left and middle panels) or skeletonized reconstructions (right panel). High magnification images shown in the middle panel correspond to the respective boxed regions in the left panel. White arrows indicate self-crossings. Scale bars: 50 μm (left panel) and 10 μm (middle panel). **(B)** Quantification of number of branches, total dendrite length, total area of dendritic arbor, and number of self-crossing per 100 μm^2^ of dendrite area of labeled mCherry-expressing MEA6^F/F^ (*n* = 5 from 5 mice) and P-Cre;MEA6^F/F^ (*n* = 5 from 5 mice) PCs. Number of branches: 776 ± 63 per cell (MEA6^F/F^) and 845 ± 70 per cell (P-Cre;MEA6^F/F^), unpaired *t*-test, *p* > 0.05. Total dendrite length: 6.0 ± 0.6 mm (MEA6^F/F^) and 6.5 ± 0.5 mm (P-Cre;MEA6^F/F^), unpaired *t*-test, *p* > 0.05. Total area: 2.2 ± 0.2 E4 μm^2^ (MEA6^F/F^) and 2.4 ± 0.2 E4 μm^2^ (P-Cre;MEA6^F/F^), unpaired *t*-test, *p* > 0.05. Number of crossing: 0.9 ± 0.2 per 100 μm^2^ (MEA6^F/F^) and 2.5 ± 0.3 per 100 μm^2^ (P-Cre;MEA6^F/F^), unpaired *t*-test, ^*^p < 0.05. Gray dots indicate individual data points. **(C)** PCs from MEA6^F/F^ and N-Cre;MEA6^F/F^ mice (P21) are shown by z-projections of confocal images (left and middle panels) or skeletonized reconstructions (right panel). High magnification images shown in the middle correspond to the respective boxed regions in the left. White arrows indicate self-crossings. Scale bars: 50 μm (left panel) and 10 μm (middle panel). Bar graphs show the quantification of number of branches, total dendrite length, total area of dendritic arbor, and number of self-crossing per 100 μm^2^ of dendrite area of MEA6^F/F^ (*n* = 9 from 9 mice) and N-Cre;MEA6^F/F^ (*n* = 5 from 9 mice) PCs. Number of branches: 752 ± 77 per cell (MEA6^F/F^) and 639 ± 112 per cell (N-Cre;MEA6^F/F^), unpaired *t*-test, *p* > 0.05. Total dendrite length: 6.0 ± 0.5 mm (MEA6^F/F^) and 4.4 ± 0.3 mm (N-Cre;MEA6^F/F^), unpaired *t*-test, ^*^p < 0.05. Total area: 2.4 ± 0.3 E4 μm^2^ (MEA6^F/F^) and 1.4 ± 0.3 E4 μm^2^ (N-Cre;MEA6^F/F^), unpaired *t*-test, ^*^p < 0.05. Number of crossing: 1.0 ± 0.2 per 100 μm^2^ (MEA6^F/F^) and 2.6 ± 0.3 per 100 μm^2^ (N-Cre;MEA6^F/F^), unpaired *t*-test, ^∗∗^*p* < 0.01. Gray dots indicate individual data points. **(D)** Protein levels of γ-Pcdh, BDNF, TrkB, Slit2, Robo2, MEA6, and PSD95 in the MEA6^F/F^ and P-Cre;MEA6^F/F^ cerebellum at P20 were analyzed by Western blotting. The results were obtained from 3 pairs of mice. β-tubulin was used as the loading control. Gray dots indicate individual data points. ^*^p < 0.05.

Thus, a subsequent question is how PC deletion of MEA6 affects motor learning? It has been shown that neurons usually develop non-overlapping patterns by self-avoidance, an active process involving contact-dependent recognition and repulsion between neighboring sister branches (Grueber and Sagasti, 2010). Interestingly, the disruption in such self-avoidance is sufficient to produce a change in motor learning (Gibson et al., 2014). Therefore, we investigated if the motor behavior alteration in P-Cre;MEA6^F/F^ mice is due to the self-avoidance of PC dendrites. To do so, we injected recombinant SFV expressing mCherry (SFV-mCherry) into the cerebellar midline of MEA6^F/F^ and P-Cre;MEA6^F/F^ mice at P21 (Jia et al., 2017), when PCs have developed nearly mature arbor morphology. This approach allowed us to visualize the entire dendritic arbor of single PCs because of the sparse expression of SFV (Jia et al., 2017). Single labeled PCs were imaged by confocal microscopy, traced and reconstructed from thin optical sections using Imaris software. In MEA6^F/F^ mice, mCherry+ PC arbors consisted of smooth primary dendrites and spiny secondary branches restricted to and arborizing within ML (Figure 5A). These dendrites showed a strong tendency of self-avoidance with merely few crosses of sister dendrites (Figure 5A). In contrast, PCs with MEA6 deletion revealed extensive overlapping of sister branches (Figure 5A). The overlaps were mainly observed between high-order spiny branches and not between smooth primary dendrites (Figure 5A). By randomly selecting 100 μm^2^ regions, which altogether cover ∼20% of each arbor (Gibson et al., 2014), we found that MEA6^F/F^ PCs have only 0.9 ± 0.2 crosses per 100 μm^2^ of dendrite length whereas P-Cre;MEA6^F/F^ PCs have a significantly higher frequency of self-crossing with 2.5 ± 0.3 per 100 μm^2^ (Figure 5B). Meanwhile, the number, total length, and total area of branches kept intact in P-Cre;MEA6^F/F^ compared with MEA6^F/F^ mice (Figure 5B). In addition, it was of interest to examine if abnormal self-avoidance of PC dendrites also occurs in N-Cre;MEA6^F/F^ mice. We injected SFV expressing GFP (SFV-GFP) into the cerebellum to visualize the dendritic arbor of single PCs in MEA6^F/F^ and N-Cre;MEA6^F/F^ mice at P21. On one hand, PCs of N-Cre;MEA6^F/F^ mice displayed extensive overlapping of sister branches (Figure 5C). On the other hand, total length and area of PC branches were decreased in N-Cre;MEA6^F/F^ mice (Figure 5C). These results from P-Cre;MEA6^F/F^ and N-Cre;MEA6^F/F^ mice indicate that MEA6 is critical to self-avoidance of PC dendrites.

A number of cell-surface molecules have been identified to be required cell-autonomously for establishing non-overlapping dendrites in a 2D or 3D plane in mammals, including γ-Pcdh (Lefebvre et al., 2012), secreted molecule Slit2 and its receptor Robo2 (Gibson et al., 2014). Thus, we measured the protein expression of γ-Pcdh, Slit2, and Robo2, as well as PSD95, BDNF, and its receptor TrkB, which are responsible for the synaptogenesis and morphogenesis of PCs, respectively (Poo, 2011; Park and Poo, 2013; Choo et al., 2017). We found that the expressionlevels of γ-Pcdh, Robo2, BDNF, TrkB, and PSD95 were not changed by PC deletion of MEA6. In contrast, the expression of Slit2 was significantly decreased in P-Cre;MEA6^F/F^ mice compared with MEA6^F/F^ mice (Figure 5D). These results implicate that MEA6 deletion causes the self-crossing of dendrites via the downregulation of Slit2.

### Slit2 Transport Is Interrupted in P-Cre;MEA6^F/F^ Mice

**FIGURE 6:**
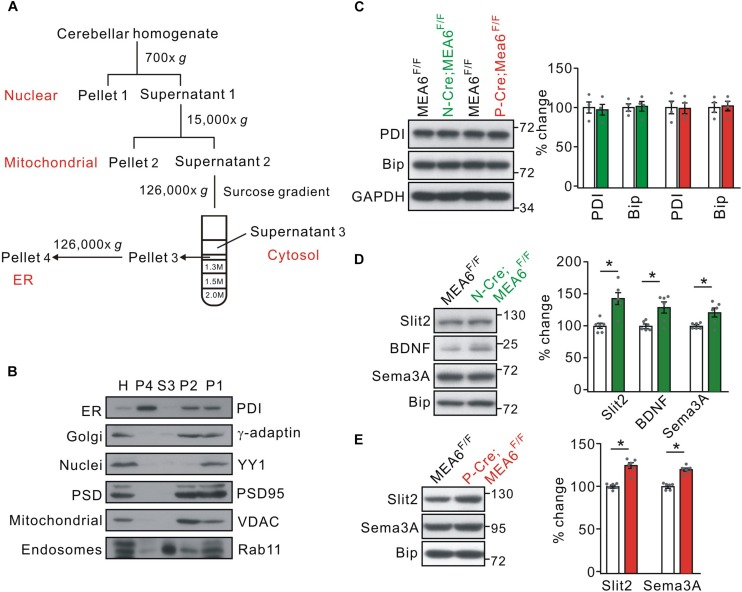
MEA6 deficiency affects the transport of Slit2 from ER to Golgi apparatus. **(A)** A cartoon illustrating the procedures for the purification of subcellular organelles. More details are given in Experimental Procedures. **(B)** The purification of ER was confirmed by the Western blotting assay of marker proteins, including PDI, γ-adaptin, YY1, PSD95, VDAC, and Rab11. H, P1, P2, S3 and P4 refer to homogenate, pellet 1, pellet 2, supernatant 3, and pellet 4, respectively. The experiment was performed using 3 WT mice. **(C)** The protein levels of PDI and Bip were not changed in both N-Cre;MEA6^F/F^ and P-Cre;MEA6^F/F^ mice compared with corresponding control MEA6^F/F^ mice. GAPDH was used as the loading control. The results were obtained from 4 pairs of mice. Gray dots indicate individual data points. **(D)** Western blotting assay of Slit2, BDNF, and Sema3A in ER purified from MEA6^F/F^ and N-Cre;MEA6^F/F^ mice cerebellum at P21. Bip was used as the internal control. Slit2: 100 ± 4% (MEA6^F/F^) and 140 ± 11% (N-Cre;MEA6^F/F^). BDNF: 100 ± 2% (MEA6^F/F^) and 130 ± 10% (N-Cre;MEA6^F/F^). Semaphorin 3A (Sema3A): 100 ± 2% (MEA6^F/F^) and 121 ± 8% (N-Cre;MEA6^F/F^). The results were obtained from 6 pairs of mice. Gray dots indicate individual data points. Unpaired *t*-test, ^*^p < 0.05. **(E)** Western assay of Slit2 and Sema3A in ER purified from MEA6^F/F^ and P-Cre;MEA6^F/F^ mice cerebellum at P21. Bip was used as internal control. Slit2: 100 ± 1% (MEA6^F/F^) and 122 ± 4% (P-Cre;MEA6^F/F^). Sema3A: 100 ± 1% (MEA6^F/F^) and 120 ± 2% (P-Cre;MEA6^F/F^). The results were obtained from 6 pairs of mice. Gray dots indicate individual data points. Unpaired *t*-test, ^*^p < 0.05.

Using tandem mass tag technique, Zhang et al. (2018) show that MEA6 is involved in protein trafficking between ER and Golgi apparatus in cultured cortical neurons, which might explain the reduced expression of Slit2 in P-Cre;MEA6^F/F^ mice. We therefore inspected whether MEA6 ablation affects the forward protein transport in the cerebellum, taking advantage of a series of centrifugations to purify organelles (Figure 6A; also see Hammond et al., 2012). The purification of ER fraction was confirmed with distinct organelle markers, PDI (ER), γ-adaptin (Golgi apparatus), YY1 (nuclei), PSD95 (synapse), VDAC (mitochondria), and Rab11 (endosome) (Figure 6B). We first examined whether ER was affected by MEA6 deficiency by comparing the marker molecules for ER, PDI and Bip (Haefliger et al., 2011; Shimizu et al., 2017; McLelland et al., 2018). Our results indicated that both molecules were not changed in N-Cre;MEA6^F/F^ and P-Cre;MEA6^F/F^ mice compared with corresponding control mice (Figure 6C), suggesting that ER is not affected by PC deletion of MEA6. Second, we compared the subcellular expression of a number of secretary molecules, including Slit2, BDNF, and Semaphorin 3A between MEA6^F/F^ and N-Cre;MEA6^F/F^ mice. Interestingly, our results showed that the expression levels of these secretary molecules increased in ER fraction, which was indexed by the expression of Bip (Figure 6D). Finally, we compared the subcellular expression of Slit2 and Semaphorin 3A between MEA6^F/F^ and P-Cre;MEA6^F/F^ mice. Similar to the results obtained from N-Cre;MEA6^F/F^ mice, we found that the expression levels of Slit2 and Semaphorin 3A significantly increased in the ER fraction (Figure 6E). These results indicate that the deletion of MEA6 tethers secretary molecules in ER and then decreases their expression.

## Discussion

Meningioma expressed antigen 6 is important for the secretion of proteins including collagen, VLDL, and insulin (Saito et al., 2011, 2014; Wang et al., 2016; Fan et al., 2017). It also regulates the transport of cellular components in neurons, and its deficiency causes abnormal development in cerebral cortex (Zhang et al., 2018). In the present work, we provide new evidence for the functions of MEA6 in cerebellar development and motor behaviors by generating conditional knockout mice. We found that both N-Cre;MEA6^F/F^ and P-Cre;MEA6^F/F^ mice exhibit defects in cerebellar development and motor performance. N-Cre;MEA6^F/F^ mice had a shrunken cerebellum and lobular layers and impaired gait behavior at P25, while P-Cre;MEA6^F/F^ mice displayed extensive self-crossings of PC dendrites and impaired motor learning. It was apparent that the deletion of MEA6 driven by Nestin-Cre produced more profound influence on the development and the function of the cerebellum, suggesting that MEA6 deletion may affect other cells except PCs in the cerebellum. A phenotype, which both conditional knockout mice shared in common, was that the transport of Slit2, BDNF and Semaphorin 3A was interrupted and thus these proteins were tethered in ER. Combined with previous work showing impaired protein transport from ER to Golgi apparatus and the fragmentation of Golgi structure in cultured cortical neurons (Zhang et al., 2018), our result might explain why the development of PCs and the cerebellum was impaired in these conditional knockout mice. In addition, we found that Slit2 in cerebellar extracts of P-Cre;MEA6^F/F^ mice was reduced, which might be ascribed to the retention of Slit2 in ER. Similar findings have been reported, i.e., total expression of AMPA receptors (AMPARs) is reduced because AMPARs are trapped in ER by Stargazin knockout (Tomita et al., 2003). Based on our findings showing the essential roles of MEA6 on the development and function of the cerebellum at early stage, it can be deduced that the absence or the loss-of-function of MEA6 may lead to severe neurodegeneration not only in the cerebellum but also in whole brain. Therefore, our study provides more insights into the pathogenesis of Fahr’s syndrome, which includes neurological and movement disorders (Oliveira et al., 2007) and may be associated with MEA6 mutation (Lemos et al., 2011).

Meningioma expressed antigen 6 regulates ER-Golgi apparatus trafficking through the regulation of COPII complex formation in secretary cells and neurons (Saito et al., 2011, 2014; Wang et al., 2016; Fan et al., 2017; Zhang et al., 2018). COPII is composed of a small GTPase (SAR1) and coat proteins (Antonny and Schekman, 2001; Lee and Miller, 2007; Miller and Schekman, 2013). Zhang et al. (2018) demonstrate that MEA6 together with SAR1 is enriched in neuronal dendritic branch and the deletion of MEA6 in neurons alters the proper function of COPII through the disruption of SAR1 activity, which results in the downregulation of postsynaptic molecules critical to dendritic outgrowth and synaptic formation, such as PSD95, TrkB, CamKII, and membrane receptors and ion channels. In the present work, we found that MEA6 ablation reduced the trafficking and expression of several secretary proteins, such Slit2, BDNF, and Semaphorin 3A, but had no effects on γ-Pcdh, Robo2, TrkB, and PSD95, implicating that MEA6 deficiency has more influence on secretary proteins but not membrane proteins. This inconsistency with previous work may be due to the different regions of brain observed in each study, although the present results were closer to those findings obtained from secretary cells (Saito et al., 2011, 2014; Wang et al., 2016; Fan et al., 2017). Future study of MEA6 in neurons would need to define its biological functions on protein trafficking.

It has been discovered that two molecular pathways, γ-Pcdh and Slit2/Robo2, independently control self-recognition and self-avoidance of PC dendritic branch (Lefebvre et al., 2012; Gibson et al., 2014). We found that MEA6 ablation in PCs impaired Slit2 trafficking from ER to Golgi apparatus and reduced the expression of Slit2. In comparison, the transport and expression of Robo2 and γ-Pcdh were not affected. The impaired self-avoidance of PC dendrites in P-Cre;MEA6^F/F^ mice may be caused by the reduced expression of Slit2, because the knockout of either Slit2 or Robo2 is sufficient to induce self-crossings in PCs (Gibson et al., 2014). Meanwhile, P-Cre;MEA6^F/F^ mice displayed slight defective in motor learning without any alteration in gait, consistent with previous finding that Robo2, but not Slit2, in PCs is associated with gait alterations (Gibson et al., 2014). In summary, our results confirm the critical roles of Slit2/Robo2 signaling in self-avoidance of PC dendrites and motor performance. Little is known about the consequences of disrupting self-avoidance for circuit function or animal behavior, although the importance of local molecular interactions during dendritic development has been highlighted (Jan and Jan, 2010). PC dendritic branch is critical for integrating diverse synaptic inputs (Häusser et al., 2000) and normal motor behavior (Donald et al., 2008; Becker et al., 2009; Li et al., 2010; Sergaki et al., 2010). Interestingly, self-avoidance defect only occurs in spiny distal branches of parallel fiber, but not climbing fibers that only innervate with primary dendrites (Gibson et al., 2014), suggesting that the alteration in parallel fiber-PC innervations may underlie such motor deficits. We hypothesize that MEA6 may alter synapse distribution across branches or their connectivity transmission and further change the patter of neural circuits, which needs further investigation by electron microscopy and electrophysiology.

## Conclusion

The deletion of MEA6 causes defects in cerebellar development and motor performance, including shrunken lobules, extensive self-crossings of PC dendrites, abnormal gait and motor learning. All these phenotype may be associated with impaired transport of secretary proteins from ER to Golgi apparatus.

## Ethics Statement

All experiments were approved by the Animal Experimentation Ethics Committee of Zhejiang University and specifically designed to minimize the number of animals used.

## Author Contributions

X-TW and YS designed the research and wrote the manuscript. X-TW, X-YC, F-XX, LZ, RZ, and K-YM performed the research. Z-HX provided the unpublished tools and techniques. X-TW, X-YC, and YS analyzed the data. All authors have read and approved the final manuscript.

## Conflict of Interest Statement

The authors declare that the research was conducted in the absence of any commercial or financial relationships that could be construed as a potential conflict of interest.

The handling Editor is currently editing co-organizing a Research Topic with one of the authors YS, and confirms the absence of any other collaboration.
